# Residual posttraumatic stress disorder symptoms after provision of brief behavioral intervention in low‐ and middle‐income countries: An individual‐patient data meta‐analysis

**DOI:** 10.1002/da.23221

**Published:** 2021-11-09

**Authors:** Aemal Akhtar, Phiona Koyiet, Atif Rahman, Alison Schafer, Syed Usman Hamdani, Pim Cuijpers, Marit Sijbrandij, Richard A. Bryant

**Affiliations:** ^1^ Clinical, Neuro and Developmental Psychology, World Health Organization Collaborating Centre for Research and Dissemination of Psychological Interventions Vrije Universiteit Amsterdam The Netherlands; ^2^ School of Psychology University of New South Wales Sydney Australia; ^3^ World Vision International Nairobi Kenya; ^4^ University of Liverpool Liverpool England; ^5^ World Health Organization Geneva Switzerland; ^6^ Human Development Research Foundation Islamabad Pakistan

**Keywords:** behaviour therapy, posttraumatic stress disorder, problem management plus, randomised controlled trial, residual symptoms, task‐sharing

## Abstract

**Background:**

To address shortages of mental health specialists in low‐ and middle‐income countries, task‐shifting approaches have been employed to train nonspecialists to deliver evidence‐based scalable psychosocial interventions. Problem Management Plus (PM+) is a brief transdiagnostic nontrauma focused intervention for people affected by adversity. This study reports on the capacity of PM+ to address specific symptoms of posttraumatic stress disorder (PTSD).

**Methods:**

Individual patient data from three randomised controlled trials were combined and analysed to observe the impacts of PM+ (*n *= 738) or enhanced treatment as usual (ETAU) (*n *= 742) interventions on specific PTSD symptoms at posttreatment and 3‐month follow‐up. The PTSD‐Checklist for DSM‐5 (PCL‐5) was used to index PTSD symptoms, and presence of each symptom was defined as moderate severity (score ≥ 2 on individual items).

**Results:**

The average PCL‐5 score at baseline was 26.1 (SD: 16.8) with 463 (31.3%) scoring above 33, indicative of a diagnosis of PTSD. Following intervention, 12.5% and 5.8% of participants retained a score greater than 33 at postassessment and follow‐up, respectively. There was greater symptom reduction for PM+ than for ETAU for most symptoms. Hyperarousal symptoms were the most common residual symptoms after PM+, with more than 30% of participants reporting persistent sleep disturbance, concentration difficulties, and anger.

**Conclusion:**

PM+ led to greater reduction in symptoms relating to re‐experiencing and avoidance. The evidence indicates that strategies focusing on hyperarousal symptoms including sleep, concentration, and anger difficulties, could be strengthened in this brief intervention.

## 1 INTRODUCTION

People affected by war, conflict, and humanitarian crises disproportionately live in low‐ and middle‐income countries (LMICs), and they are at increased risk for psychological problems (Barbui & Tansella, 2013; Charlson et al., 2019). This is not surprising because these vulnerable populations can be exposed to significant adversity, including poverty, sexual violence, torture, war, and displacement. Despite the prevalence of mental health problems in LMICs, including people living in humanitarian contexts, it is estimated that at least 90% of adults with mental health needs do not receive minimum adequate care (Chisholm et al., 2016). Programs exist that can address mental health conditions in these typically poor resource settings (Morina et al., 2017a, 2017b), but they are often not implemented because (a) these interventions tend to only target a single diagnostic outcome (e.g., posttraumatic stress disorder (PTSD; (Neuner et al., 2008)), (b) are generally resource and time intensive (Bolton et al., 2007), and (c) often require mental health specialists who are lacking in LMICs (Bass et al., 2013). These factors preclude many LMICs and humanitarian responses from implementing much‐needed mental health services that their populations require (Patel et al., 2018; Tol et al., 2012).

This situation has led to increased recognition that addressing the mental health and psychosocial needs of people in humanitarian crisis settings requires transferring a portion of mental health delivery to trained and supervised nonspecialist workers. This shift is appealing in low resource settings because it overcomes the limitation of the scarcity of mental health specialists, reduces costs, and can minimize stigma associated with specialist mental health care (Patel et al., 2018). Increasingly, programs have utilized these “task‐shifting” approaches in which local providers who lack formal mental health qualifications are trained to deliver structured manualized interventions for common mental disorders. One meta‐analysis found these interventions are moderately effective in LMICs (Singla et al., 2017).

The World Health Organization (WHO) has recently adopted this approach by developing a range of mental health interventions that are intended to address common mental disorders (i.e., anxiety and depression) in populations affected by adversity. Inherent in this approach is that these interventions are brief, affordable, can be easily trained to nonspecialists, and in this manner can be scaled up to achieve maximum reach in an LMIC and with limited resources. The first program of this type developed by the WHO was titled Problem Management Plus (PM+), a transdiagnostic intervention that involves five sessions that teach people skills that have been shown to be the most effective in reducing common mental disorders in controlled trials (Dawson et al., 2015). Specifically, PM+ teaches nonspecialist providers to train people with common mental disorders skills in arousal reduction, problem‐solving, behavioral activation, and strengthening social supports. PM+ delivered to individuals has been shown to be effective in reducing psychological distress in adverse settings. The two large trials comprised women affected by gender‐based violence in Kenya (Bryant et al., 2017) and men and women in in a conflict‐affected region in Pakistan (Rahman et al., 2016). Additionally, PM+ has been delivered in a group format in Pakistan, which has also been shown to be effective relative to treatment as usual (Rahman et al., 2019).

Despite the overall efficacy of PM+ in reducing psychological distress and symptoms of common mental disorders, there is a need to better understand how it impacts specific psychological symptoms following adversity, such as the experiences of people who have lived through interpersonal trauma or humanitarian crises. One treatment study of female veterans with PTSD that employed trauma‐focused cognitive behaviour therapy (TF‐CBT) found that hyperarousal symptoms, and especially sleep problems and irritability, were most resistant to change with treatment (Schnurr & Lunney, 2019). This finding accords with a trial of female rape survivors with PTSD, and found that sleep problems were most resistant to TF‐CBT (Larsen et al., 2019). The finding that many people continue to experience functional difficulties following remission of PTSD diagnosis (Bryant et al., 2016) points to the need to better understand psychological difficulties that persist after treatment. Although some inroads have been made in relation to trauma‐focused psychotherapies, there is a need to understand how scalable interventions, such as PM+, address specific PTSD symptoms in humanitarian and development contexts where the prevalence of PTSD is elevated (Charlson et al., 2019). This is relevant for PM+ because although it is not a trauma‐focused intervention, previous trials have shown that PM+ leads to significant reductions in PTSD severity, in adversity‐affected LMICs and conflict settings (Rahman et al., 2016, 2019). In both situations, many people in these category do suffer from PTSD, or experience events that could lead to PTSD. In turn, understanding the residual symptoms of PTSD following PM+ would provide information on how the intervention reduces PTSD symptoms as well as inform the development of future scalable interventions.

To this end, this study conducts an individual‐patient data meta‐analysis (IPD‐MA) of the three large controlled trials that have been published to determine which symptoms of PTSD are most persistent after provision of PM+ (Bryant et al., 2017; Rahman et al., 2016, 2019). It further aims to inform the potential of PM+ to be a useful brief intervention across humanitarian and adversity settings where PTSD is considered one of the common mental disorders experienced.

## 2 METHODS

### 2.1 Materials and methods

This IPD analysis focused on prospectively registered randomized clinical trials (RCT) of individual PM+ in Pakistan ((Rahman et al., 2016), ACTRN12614001235695) and Kenya ((Bryant et al., 2017), ACTRN12616000032459), and group PM+ in Pakistan ((Rahman et al., 2019), ACTRN12616000037404). Details about the original studies have been published elsewhere (Chiumento et al., 2017; Sijbrandij et al., 2015, 2016). Initial studies of the PM+ manual required approval of their research protocols through the WHO Ethical Review Committee in addition to the relevant governing bodies in countries that the trials were conducted. All participants provided written informed consent before taking part in the trials.

### 2.2 Context and participants

Between November 2014 and August 2016 three RCTs were conducted to evaluate the effectiveness of PM+ in reducing the levels of psychological distress experienced by participants. A secondary outcome of each trial included PTSD symptoms. Data from these studies were combined into a single data set.

The study characteristics are presented in Table 1. In the Pakistan individual PM+ study, participants (*N *= 346) were recruited from three primary health care centres in the conflict‐affected urban city of Peshawar (Rahman et al., 2016). Participants received clinical assessments from their physicians; participants who were deemed to be suffering from psychological distress were referred to the study. The Pakistan group‐based PM+ study was conducted in two rural council districts of Swat (Rahman et al., 2019), and adult women (*N *= 612) were randomly screened in the community and invited into the study if they screened positively for psychological distress. The Kenya study recruited female participants (*N *= 522) through random sampling in peri‐urban neighbourhoods of Nairobi, of whom 81% had experienced gender‐based violence (Sijbrandij et al., 2016). In all three studies the initial screening procedure used the General Health Questionnaire‐12 (GHQ‐12; (Goldberg & Williams, 1988; Minhas & Mubbashar, 1996)) to identify psychological distress and the WHO‐Disability Assessment Schedule 2.0 (WHODAS 2.0), which is a 12‐item measure of general functioning (WHO, 2010). The common inclusion criteria across all three studies were (i) adults aged 18–60 years, (ii) score of ≥3 on the GHQ‐12, and (iii) score of ≥17 on the WHODAS 2.0. The exclusion criteria were identical across all three studies: (i) imminent risk for suicide, (ii) severe mental illness (e.g., psychotic disorders, substance use disorders), and (iii) significant cognitive and neurological impairment (e.g., severe intellectual disability).

**Table 1:** Study characteristics

Studies	Study design	Country	Recruitment	Inclusion criteria	Intervention	Control group	Follow‐up (weeks)	*N* (*N*_int_)	Outcome
Bryant et al. (2017)	RCT	Kenya	Community screening	Age = 18–60, GHQ ≥ 3; WHODAS ≥ 17	PM+	ETAU	6 (T_1_), 18 (T_2_)	T_0_ = 522 (260)	PCL‐5
								T_1_ = 419 (205)	
								T_2_ = 389 (191)	
Rahman et al. (2016)	RCT	Pakistan	Primary health care centres	Age = 18–60, GHQ ≥ 3; WHODAS ≥ 17	PM+	ETAU	6 (T_1_), 18 (T_2_)	T_0_ = 346 (172)	PCL‐5
								T_1_ = 137 (60)	
								T_2_ = 306 (146)	
Rahman et al. (2019)	cRCT	Pakistan	Community screening	Age = 18–60, GHQ ≥ 3; WHODAS ≥ 17	gPM+	ETAU	6 (T_1_), 18 (T_2_)	T_0_ = 612 (306)	PCL‐5
								T_1_ = 598 (298)	
								T_2_ = 578 (288)	

Abbreviations: cRCT, clustered randomized controlled trial; ETAU, enhanced treatment as usual; GHQ, General Health Questionnaire; gPM+, Group Problem Management Plus; *N*, number of participants; *N*_int_, number of participants randomized into intervention arm; PCL‐5, PTSD checklist for DSM‐5; PM+, problem management plus; RCT, randomized controlled trial; T_0_, Entry into the study (0 weeks); *T*_1_, 6‐week follow up, *T*_2_, 18‐week follow up; WHODAS, World Health Organization Disability Adjustment Scale 2.0.

### 2.3 Interventions

#### 2.3.1 Problem Management Plus

PM+ integrates problem‐solving and behavioural activation techniques that are amenable to low‐intensity delivery and are evidence‐based (Bennett‐Levy & Farrand, 2010; Cuijpers et al., 2007; Farchione et al., 2012; van't Hof et al., 2011). The individual format is delivered over five weekly sessions of 90 min duration; the group format is delivered over five weekly sessions of 120 min duration. Clients are taught: (i) arousal reduction; (ii) problem management; (iii) behavioural activation, and (iv) skills to strengthen social support. The PM+ programme has been made available publicly by the WHO (WHO, 2016). Facilitators received 8 days of training in basic counselling skills and the delivery of the PM+ intervention. For the group modality, facilitators are additionally taught group facilitation skills. Following training, the PM+ providers are required to complete two practice cases whilst attending weekly group supervision sessions focused on fidelity to the intervention, challenges faced, and helpers self‐care.

#### 2.3.2 Enhanced treatment as usual (ETAU)

The ETAU arms of all three studies included psychoeducation and referral information for locally available resources for health, vocational training, and other relevant services. Participants were provided with individual, verbal feedback regarding the results of the assessments, brief psychoeducation and referral services to primary health care centres in both Kenya and the Swat trial, and participants met with a primary healthcare physician at least once where they received assessment feedback and a psychoeducation session in the Peshawar study.

### 2.4 Measure

PTSD symptoms were measured using the PTSD Checklist for DSM‐5 (PCL‐5) (Weathers et al., 2013). The PCL‐5 is a 20‐item checklist corresponding to the 20 symptoms for PTSD as defined in DSM‐5. There are five items corresponding to intrusion symptoms, two to avoidance symptoms, seven to negative alterations in cognitions and moods, and six to alterations in arousal and reactivity. Items are rated on a 5‐point Likert scale (0 =* not at all*, 4 =* extremely*) and total scores are obtained by summing the responses for individual items (range: 0–80). Across all three studies, the PCL‐5 was adapted to ask for symptoms in the past week (instead of month) to enhance sensitivity to change. The PCL‐5 measure was adapted in all three studies in accordance with gold‐standard translation practices, with each item being translated and back‐translated by accredited translators (Bontempo, 1993). In Kenya, items were translated into Kiswahili and in Pakistan, items were translated into Urdu. The comprehensibility of the adapted versions were piloted in feasibility trials which took place before the RCTs (Dawson et al., 2016; Rahman et al., 2016). The psychometric properties of the PCL‐5 have been widely studied and has been shown to have high levels of diagnostic accuracy and internal consistency (Blevins et al., 2015; Bovin et al., 2016), including in LMIC (Mughal et al., 2020). Symptoms were rated as present if the respondent indicated that they were at least moderately bothered by them, as indicated by a score ≥2 on individual items (Weathers et al., 2013). Symptom retention was defined as the conditional probability of retaining a symptom at posttreatment and 3‐month follow‐up compared to before receiving treatment. A probable diagnosis of PTSD is made if the total score on the PCL‐5 was ≥33 (Weathers et al., 2013).

### 2.5 Statistical analysis

Means and SD are reported for continuous variables while frequencies and percentages are reported for categorical variables. Missing outcome data at postintervention and follow‐up assessments were estimated using multiple imputation (MI); data were assumed to be missing‐at‐random. As the PCL‐5 results are scored on a Likert scale, MI was conducted by chained equations using ordered logistic regression (mi impute chained ologit in Stata). We analysed the effects of PM+ versus ETAU on the conditional probability that participants retained symptoms following intervention using a multilevel logistic regression, with random effects for each trial; separate models were run for each of the 20 PTSD symptoms. A complete‐case sensitivity analysis was conducted to explore robustness of results from the MI using only participants who completed the post and follow‐up assessments. Results are reported as the estimated odds ratio (OR) and the 95% confidence intervals (CI) for each symptom are presented. All analyses were conducted using Stata 13 (StataCorp., 2013).

## 3 RESULTS

Descriptive information about study participants is presented in Table 2. In total, 1480 participants were randomised across the three trials, with 738 receiving the PM+ intervention and 742 receiving ETAU. There were 1154 (78.0%) participants who completed the postintervention assessment and 1,273 (86.0%) who completed the 3‐month follow‐up assessments. The average age of the study participants was 35.31 years (SD = 11.84). The studies comprised 1407 women (95%).

**Table 2:** Demographic and clinical characteristics of the study population (*n *= 1480)

	Kenya (*N *= 522)		Pakistan (*N *= 346)		SWAT (*N *= 612)		Combined (*N *= 1480)	
Randomization								
PM+, *N*	260		172		306		738	
ETAU, *N*	262		174		306		742	
Age, mean (SD)	35.95 (13.66)		33.08 (11.80)		36.26 (9.88)		35.41 (11.84)	
Education (years), mean (SD)	8.50 (3.98)		‐		‐		‐	
Education							‐	
No schooling, *N* (%)	‐		203 (58.7%)		503 (82.2%)		‐	
Primary (6 years), *N* (%)	‐		42 (12.1%)		51 (8.3%)		‐	
Middle (8 years), *N* (%)	‐		26 (7.5%)		22 (3.6%)		‐	
Matriculate (10 years), *N* (%)	‐		29 (8.4%)		18 (2.9%)		‐	
Intermediate, college and university (11–16 years), *N* (%)	‐		46 (13.3%)		15 (2.5%)		‐	
Gender, *N* (%)								
Female	522 (100)		273 (78.90)		612 (100)		1407 (95.07)	
Male	0 (0)		73 (21.10)		0 (0)		73 (4.93)	

PCL‐5, mean (SD)	PM+	ETAU	PM+	ETAU	PM+	ETAU	PM+	ETAU
Baseline	32.86 (20.10)	30.67 (19.17)	31.81 (14.34)	30.30 (14.22)	17.26 (11.04)	19.75 (11.70)	26.14 (17.21)	26.06 (16.14)
Postintervention	9.62 (12.54)	13.36 (15.74)	12.86 (10.69)	18.73 (11.11)	8.66 (9.17)	12.41 (8.29)	9.75 (10.76)	13.73 (12.03)
3‐month	7.56 (11.42)	7.41 (10.40)	11.90 (9.18)	17.72 (9.35)	8.48 (6.47)	10.92 (7.96)	9.00 (9.01)	11.52 (9.88)
PTSD diagnosis								
Baseline	126 (48.65)	115 (44.40)	77 (44.77)	77 (44.25)	27 (8.82)	41 (17.60)	230 (31.21)	233 (31.53)
Postintervention	13 (6.40)	31 (14.49)	9 (7.89)	12 (12.63)	10 (3.33)	8 (2.68)	32 (5.19)	51 (8.39)
3‐month	9 (4.71)	6 (3.02)	6 (4.11)	12 (7.50)	2 (0.69)	7 (2.41)	17 (2.72)	25 (3.85)
PTSD diagnosis retention								
Postintervention, *N* (%)	12 (12.24)	28 (28.00)	5 (9.80)	7 (15.56)	4 (14.81)	2 (5.00)	21 (11.93)	37 (20.00)
3‐month, *N* (%)	7 (7.14)	5 (5.95)	4 (6.35)	7 (10.61)	0 (0.00)	4 (11.11)	11 (5.95)	16 (8.60)

Abbreviations: ETAU, enhanced treatment as usual; PM+, problem management plus.

The average PCL‐5 score at baseline was 26.1 (SD = 16.76) while 463 (31.3%) participants scored ≥33, indicating that it was probable they meet diagnostic criteria for PTSD. There were 230 (31.21%) and 233 (31.53%) persons who scored ≥33 on the PCL‐5 at baseline randomized in the PM+ and ETAU arms, respectively. The Kenya and individual PM+ Pakistan trials had higher baseline PCL‐5 scores (31.76 and 31.05, respectively) and numbers of people with probable PTSD (46% and 45%, respectively) compared to the baseline PTSD levels in the Swat study (*M* = 18.51, 11% > 33). At postintervention assessment, 5.19% of people who received PM+ and 8.39% of people who received ETAU reported a score ≥33 and at 3‐month follow‐up, 2.72% (PM+) and 3.85% (ETAU) had a score ≥33. For those who scored ≥33 at baseline, 11.93% and 20% (postintervention), and 5.95% and 8.60% (3‐month follow‐up) maintained a score of ≥33 in the PM+ and ETAU arms, respectively.

**Figure 1:**
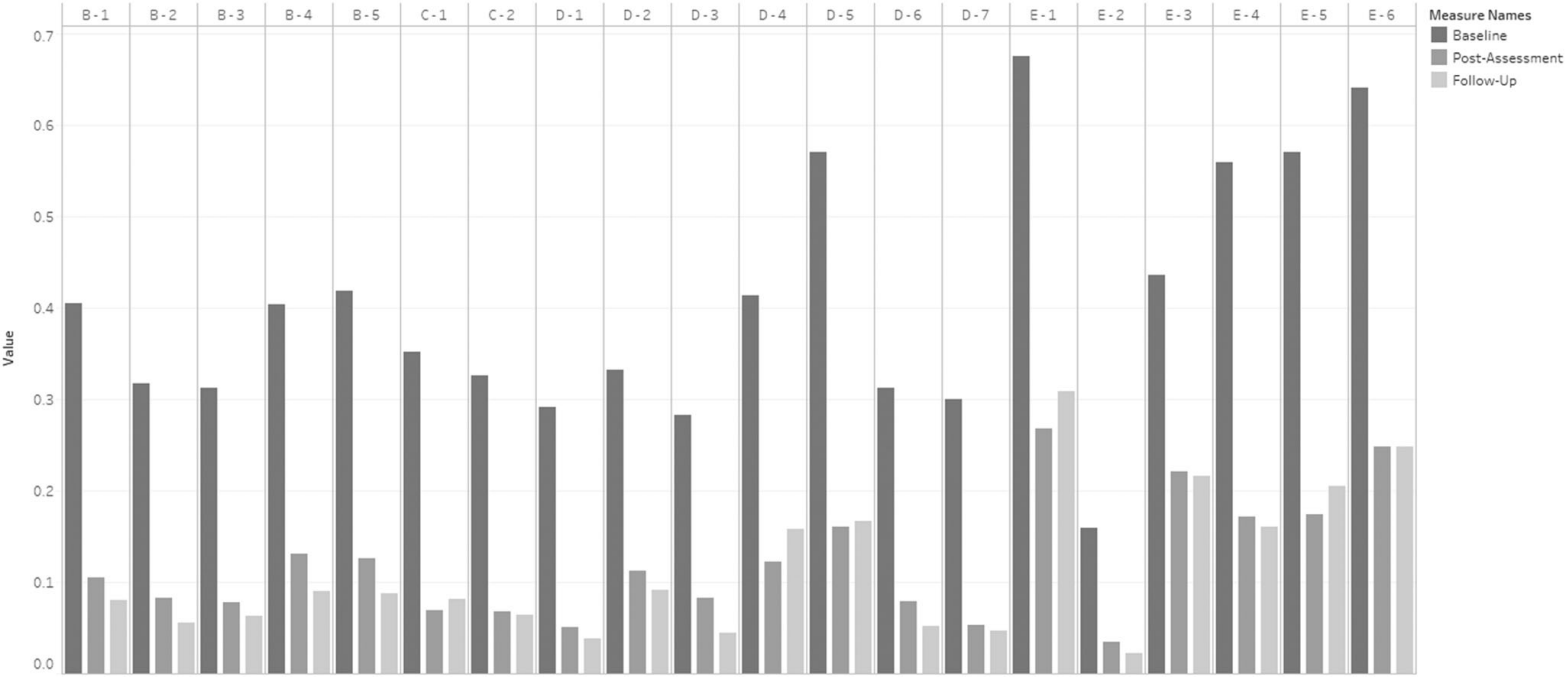
Problem management plus—symptom presence for each posttraumatic stress disorder symptom at baseline, postintervention assessment, and 3‐month follow up

Figure 1 illustrates the proportions of participants that were randomised to receive PM+ and who scored positive for symptom presence at baseline, postintervention, and 3‐month follow‐up. The most prevalent symptoms at baseline were irritability/aggression, difficulty concentrating, and sleeping (i.e., arousal symptoms). The least prevalent symptoms were inability to recall the trauma, exaggerated blame, and risky/destructive behavior. Following treatment, at both postintervention and 3‐month assessment, arousal symptoms remained the most prevalent, although the proportions of individuals who were positive for these arousal symptoms decreased markedly. Mood symptoms were among the lowest reported following PM+ at both postintervention time points, although risky behavior (an arousal symptom) was the least prevalent at all three time points.

**Figure 2:**
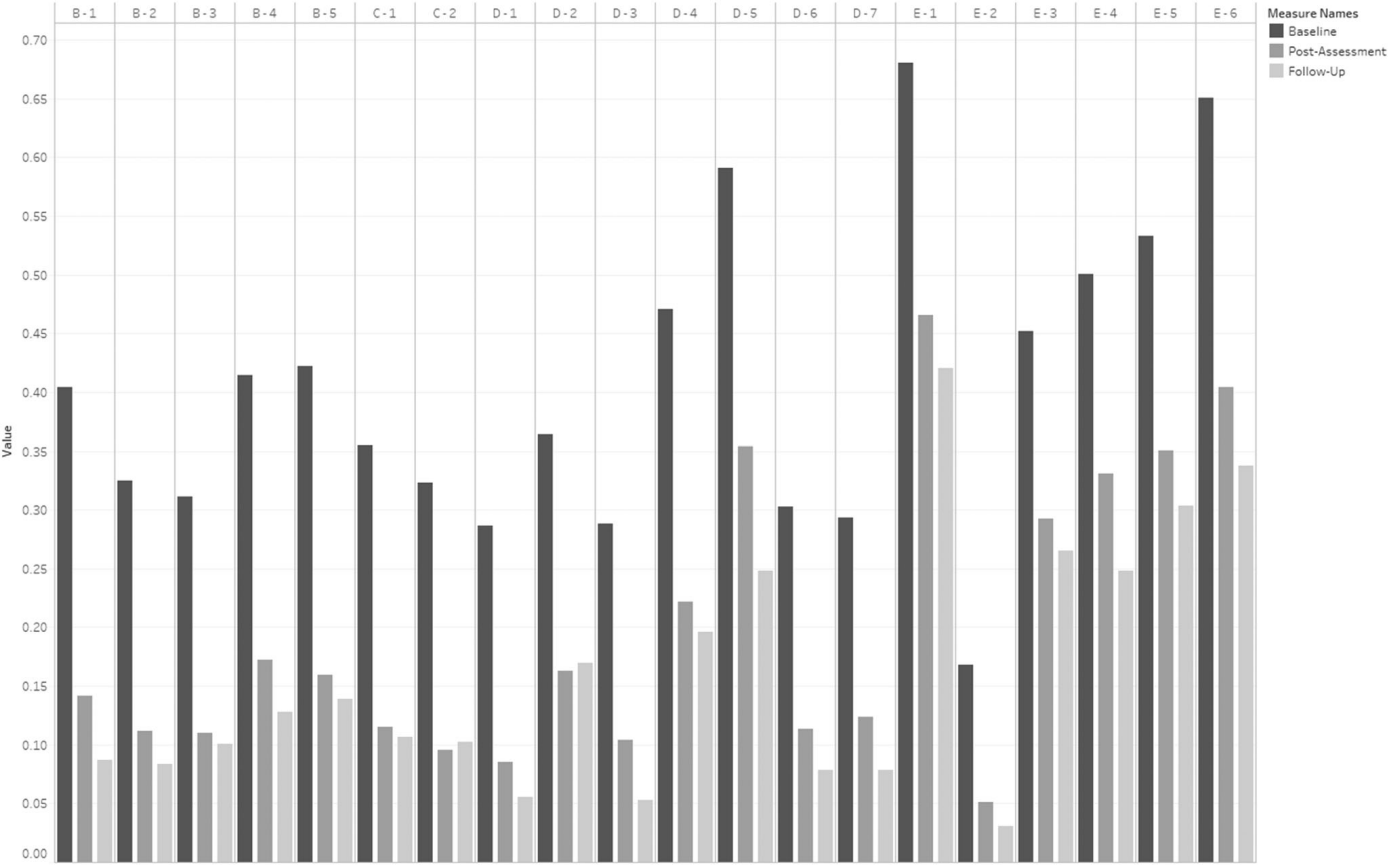
Enhanced treatment as usual—symptom presence for each posttraumatic stress disorder symptom at baseline, postintervention assessment, and 3‐month follow up

Figure 2 illustrates the prevalence of PTSD symptoms for those who received ETAU. The baseline rates were similar to participants who received PM+. Even though this group showed marked reductions of symptoms of PTSD, the prevalence of all symptoms were higher than the PM+ participants; particularly mood and arousal symptoms. This was also true at 3‐month follow‐up.

Tables 3 and 4 present the proportions of individuals who retained symptoms following intervention (Table 3) and at 3 months (Table 4); they also present the ORs and corresponding 95% CI. Across all 20 symptoms, there were higher rates of amelioration of symptoms for those who received PM+ compared to those who received ETAU, with the largest effects seen in mood and arousal symptoms. The symptoms with the greatest absolute remission following the PM+ intervention were avoidance of thoughts and behaviours. When comparing the conditional probabilities of symptom retention immediately following interventions and at 3‐month follow‐up, the ORs point estimates indicate that participants receiving the ETAU intervention had higher odds of symptom retention that those who received PM+, when compared to baseline. Specifically, the odds of symptoms amelioration were significantly higher for those who received PM+ compared to ETAU in: physical reactivity, avoidance of thoughts, avoidance of people/places, negative affect, decreased interest, difficulty with positive affect, irritability/aggression, heightened startle reaction, difficulty concentrating, and difficulty sleeping. At 3‐month follow‐up, significant reductions in symptom rates were maintained for: physical reactivity, avoidance of people/places, decreased interest, irritability/aggression, heightened startle reactions, difficulty concentrating, and difficulty sleeping. In addition, at 3‐month follow‐up, the following symptoms had significantly higher odds of being reduced in those who received PM+ compared to ETAU: negative thoughts/assumptions and hypervigilance. There were no changes in the results of the sensitivity analysis; this indicates robustness of the primary analysis strategy.

**Table 3:** Percentage of participants retaining each posttraumatic stress disorder symptom after treatment by treatment type—postassessment

	PM+		ETAU		Multiple imputation		Complete case	
Item	%	*N*	%	*N*	OR	95% CI	OR	95% CI
B–1 Intrusive memories	15.25	34	21.61	51	1.44	0.92–2.26	1.53	0.95–2.49
B–2 Nightmares	15.03	26	22.83	42	1.53	0.90–2.62	1.66	0.96–2.87
B–3 Flashbacks	14.37	37	20.56	37	1.42	0.82–2.45	1.54	0.88–2.69
B–4 Emotional distress	23.50	51	27.87	68	1.27	0.85–1.90	1.28	0.83–1.96
B–5** Physical reactivity	21.78	49	29.66	70	1.57	1.05–2.35	1.68	1.09–2.60
C–1** Avoidance of thoughts	12.89	25	22.39	45	1.89	1.13–3.17	2.04	1.18–3.52
C–2** Avoidance of people/places	11.48	21	20.99	38	2.01	1.10–3.66	2.13	1.17–3.88
D–1 Dissociative amnesia	8.39	13	15.15	25	1.75	0.93–3.31	1.96	0.96–3.99
D–2 Negative thoughts/assumptions	18.58	34	21.80	46	1.23	0.76–1.97	1.26	0.76–2.08
D–3 Exaggerated blame	17.76	27	23.35	39	1.22	0.72–2.09	1.41	0.81–2.44
D–4** Negative affect	18.58	32	33.45	92	2.07	1.38–3.10	2.33	1.51–3.57
D–5** Decreased interest	19.41	66	42.36	158	2.88	2.05–4.05	3.09	2.20–4.35
D–6 Feeling isolated	15.47	28	22.53	41	1.59	0.95–2.67	1.60	0.93–2.74
D–7** Difficulty with positive affect	9.66	17	22.35	38	2.40	1.32–4.34	2.69	1.45–4.99
E–1** Irritability/aggression	31.33	125	57.01	240	2.73	2.05–3.63	3.02	2.24–4.07
E–2 Risky/destructive behaviour	10.34	9	16.67	15	1.56	0.70–3.50	1.79	0.73–4.39
E–3 Hypervigilance	32.45	86	40.14	112	1.28	0.87–1.89	1.34	0.91–1.96
E–4** Heightened startle reaction	23.65	79	46.23	147	2.72	1.93–3.84	2.88	2.04–4.07
E–5** Difficulty concentrating	21.43	75	44.88	149	2.78	1.98–3.91	3.09	2.18–4.37
E–6** Difficulty sleeping	30.10	115	48.97	190	2.16	1.60–2.91	2.23	1.66–3.00

*Note*: Significant values are underlined.

Abbreviations: CI, confidence interval; ETAU, enhanced treatment as usual; OR, odds ratio; PM+, problem management plus.

** *p* < .05.

**Table 4:** Percentage of participants retaining each posttraumatic stress disorder symptom after treatment by treatment type—3‐month follow‐up

	PM+		ETAU		Multiple imputation		Complete case	
Item	%	*N*	%	*N*	OR	95% CI	OR	95% CI
B–1 Intrusive memories	12.72	29	13.01	32	1.03	0.61–1.74	1.02	0.59–1.75
B–2 Nightmares	9.50	17	15.08	30	1.58	0.88–2.85	1.69	0.90–3.19
B–3 Flashbacks	11.24	20	17.01	33	1.50	0.84–2.67	1.62	0.89–2.94
B–4 Emotional distress	16.29	36	18.88	47	1.22	0.77–1.93	1.21	0.75–1.96
B–5** Physical reactivity	15.42	37	24.81	66	1.73	1.12–2.68	1.85	1.18–2.91
C–1 Avoidance of thoughts	13.17	27	17.13	37	1.47	0.85–2.54	1.45	0.83–2.54
C–2** Avoidance of people/places	12.50	23	19.07	37	1.83	1.03–3.24	1.88	1.05–3.36
D–1 Dissociative amnesia	4.82	8	8.24	14	1.63	0.67–3.97	1.78	0.73–4.38
D–2** Negative thoughts/assumptions	10.05	20	20.17	47	2.29	1.31–4.00	2.38	1.34–4.23
D–3 Exaggerated blame	9.68	15	11.18	19	1.19	0.60–2.39	1.17	0.57–2.40
D–4 Negative affect	25.64	60	27.46	81	1.12	0.76–1.66	1.14	0.77–1.70
D–5** Decreased interest	20.87	72	29.63	112	1.58	1.12–2.23	1.59	1.12–2.24
D–6 Feeling isolated	9.84	18	13.44	25	1.43	0.76–2.67	1.41	0.73–2.70
D–7 Difficulty with positive affect	6.70	12	11.60	21	1.56	0.76–3.20	1.66	0.78–3.52
E–1** Irritability/aggression	36.63	152	50.23	221	1.68	1.26–2.24	1.74	1.30–2.33
E–2 Risky/destructive behaviour	3.16	3	8.49	9	1.67	0.53–5.26	2.84	0.75–10.84
E–3** Hypervigilance	26.92	70	37.41	107	1.59	1.04–2.42	1.73	1.12–2.67
E–4** Heightened startle reaction	18.42	63	34.95	115	2.33	1.61–3.37	2.53	1.73–3.68
E–5** Difficulty concentrating	23.10	82	38.19	131	2.02	1.45–2.83	2.13	1.50–3.01
E–6** Difficulty sleeping	28.32	111	41.27	175	1.74	1.29–2.34	1.81	1.34–2.45

*Note*: Significant values are underlined.

Abbreviations: CI, confidence interval; ETAU, enhanced treatment as usual; OR, odds ratio; PM+, problem management plus.

** *p* < .05.

## 4 DISCUSSION

We examined the residual symptoms of PTSD following a low‐intensity intervention across urban, peri‐urban, and rural samples in three major trials in two LMICs, Kenya and Pakistan. We found that PM+ resulted in higher alleviation of symptoms when compared to ETAU. This was most notable across avoidance, cognition/mood, and arousal symptoms. These trends were similar at both postintervention assessments and 3‐month assessments.

PM+ had a significant impact on the alleviation of avoidance symptoms when compared to ETAU immediately following the program. Moreover, there was approximately a 75% reduction of re‐experiencing symptoms (e.g., intrusive memories, nightmares, flashbacks). These are interesting findings insofar as PM+ is not a trauma‐focused intervention, and does not directly target emotional processing of trauma memories or avoidance of trauma reminders. Trauma‐focused therapies purportedly lead to reduction of PTSD symptoms because they promote emotional processing of trauma memories and associated emotions through explicit exposure to trauma memories, which can facilitate extinction learning of previously learnt association of fear (Foa, 2006). It is possible that the use of problem‐solving, behavioural activation, and social support in PM+ prompts participants to engage in activities that facilitate emotional processing because of discussion about core problems or approaching previously avoided situations that are reminiscent of the traumatic experience. It is also possible that the focus on problem‐solving skills in PM+ may address aspects of avoidant behaviors; for example, problem‐solving approaches to being reluctant to shop at a local market because it reminds a person of being at a market when it was bombed may lead to proactive strategies to return to markets, thereby implicitly engaging in in vivo exposure activities. Further, TF‐CBT is intended to involve integration of new information that corrects excessively negative appraisals about the person and their world (Ehlers & Clark, 2000). Problem‐solving and behavioural activation strategies may result in beliefs about danger of one's environment or one's inadequacy may be alleviated by these strategies, which could also reduce avoidance behaviors. The finding that the superior effect regarding avoidance thoughts was not observed at 3 months may point to the potential benefit of providing booster sessions to maintain the relative benefits of PM+.

The finding that negative cognitive and mood symptoms were better addressed by PM+ than ETAU may also be attributed to the behavioral activation components of PM+ intervention. There is considerable evidence that behavioral activation reduces low mood (Ekers et al., 2014), arguably because it prompts individuals to engage in potentially pleasurable activities that can increase their sense of reward. Behavioral activation may additionally promote self‐efficacy through successful completion of tasks, which in turn can improve self‐esteem and self‐related appraisals (Benight & Bandura, 2004). Further, the act of problem‐solving itself can promote self‐mastery because it facilitates the perception that one can influence outcomes, thereby potentially reducing learned helplessness and enhancing mood (Cassidy & Long, 1996).

At follow‐up there were fewer PM+ participants with arousal symptoms, relative to those who received ETAU, including hypervigilance, startle response, concentration difficulties, anger, and sleep disturbance. One of the core strategies of PM+ aims to reduce arousal via simple techniques, primarily deep breathing strategies. Previous research has found a number of breathing‐based techniques helpful for persons with PTSD, specifically in reducing hyperarousal symptoms (Seppälä et al., 2014). Moreover, meta‐analyses indicate that slow breathing is efficacious in reducing a range of arousal symptoms, including anger, sleep disturbance, and poor concentration (Zaccaro et al., 2018). Thus it is unsurprising that teaching participants diaphragmatic breathing during the course of PM+ led to reductions in arousal‐related problems of PTSD.

Although PM+ resulted in reductions of arousal‐related problems, hyperarousal symptoms tended to be the most resistant to remission across both arms. Consistent with prior trials that have employed trauma‐focused psychotherapy for PTSD (Larsen et al., 2019; Schnurr & Lunney, 2019), this study found that sleep problems was the most retained symptom, followed by hypervigilance, startle response, anger, and concentration difficulties 3 months after PM+. This finding across treatment trials underscores the recognition that sleep disturbance is a common transdiagnostic problem for many people with psychological distress which might assume independent status as distinct from other presenting diagnoses (Spoormaker & Montgomery, 2008; Zayfert & DeViva, 2004). Sleep impairment is a commonly persistent problem after treatment for depression (Menza et al., 2003), suggesting that lingering sleep difficulties in participants may be associated with ongoing depression. It is also worth noting that many participants in the current trials lived in harsh conditions, largely characterized by poverty, potential interpersonal threats, and other forms of adversity. Such conditions can result in arousal symptoms reflecting reactions to imminent threats rather than being symptomatic of a psychological condition. This remains a major challenge for scalable, transdiagnostic interventions given the cyclic nature of sleep impairment contributing to marked impairment in PTSD and various other mental health conditions (Harvey & Tang, 2012; Werner et al., 2019).

The reduction of symptoms for participants who received ETAU is additionally noteworthy. Several explanations may be considered to account for this pattern. First, ETAU involved providing participants with psychoeducation following their baseline assessment. On its own, this may have been beneficial, as supported by evidence that psychoeducation can reduce some PTSD symptoms (Hadar Lubin et al., 1998; Swan et al., 2017). Second, there is evidence that repeated assessments themselves may result in reduction of symptom reporting, which formed part of the methologies in these trials (Tarrier et al., 1999).

PM+ was intended to be a low‐intensity, scalable intervention allowing for task‐sharing in low‐resourced, adverse and humanitarian settings. The intervention aimed to address the mental health gap while alleviating the burden on specialized services. Additionally, it was developed with the intention of being transdiagnostic insofar as it would address symptoms across multiple common mental disorders. Having better insight into the specific symptoms impacted by the intervention will allow for more effective and targeted interventions in the future. For example, knowledge that PM+ contributes to reducing symptoms of PTSD may make this intervention more viable in postconflict humanitarian contexts. Further, understanding which symptom clusters are resistant to PM+ allows for the development and inclusion of additional psychological strategies as active components. These future developments are important as PM+ is being successfully adapted to augment treatment in specialist mental health care facilities (Hamdani et al., 2021) and also in high‐income countries with advanced health resources (de Graaff et al., 2020).

## 5 LIMITATIONS AND FUTURE RESEARCH

We note several methodological limitations. First, two of the three included studies only enrolled women, which may limit the generalizability of these findings beyond female populations. Recruitment of males can be difficult in humanitarian crisis settings (Affleck et al., 2018), however there are sex differences in both PTSD and in treatment response (Olff, 2017). Therefore, continued understanding about how gender impacts symptom remission remains important. Second, the trial participants were selected due to heightened levels of psychological distress and no formal diagnostic assessments via structured clinical interviews were conducted. Third, there was a large difference on average baseline scores as well as the number of participants who scored ≥33 on the PCL‐5 in Kenya/Peshawar and Swat. This may be attributed to the different contexts and participants selected; Kenya data was based only on women who had a reported history of gender‐based violence, while the Peshawar study recruited participants in a high‐stress environment where acute conflict was commonplace. In contrast, the Swat trial participants were recruited from rural districts affected by chronic conflict and natural disaster. In the context, we also note that there is no convergent evidence regarding the recommended cut‐off scores on the PCL‐5 to identify probable PTSD in humanitarian settings. Fourth, trials were not able to collect detailed information about usual care treatments that participants received instead of PM+. Being able to compare the ETAU interventions across studies to look at commonalities and differences would allow for a better understanding of the patterns and results observed across the three trials. Lastly, we recognize that meeting diagnostic criteria was not a prerequisite for entry into the studies.

## 6 CONCLUSION

As transdiagnostic programs are increasingly delivered by lay providers, it is important to understand how these programs impact on specific symptoms across a range of disorders. This is the first study to investigate the direct effects of PM+ on the residual symptoms on PTSD. Although PM+ was designed as a transdiagnostic intervention and is not specifically a trauma‐focused treatment, it is a valuable finding that it was still effective to reduce many core PTSD symptoms, including re‐experiencing and avoidance symptoms. Importantly, PM+ was less successful in reducing certain hyperarousal symptoms, which may reflect ongoing problems associated with living in contexts of adversity, poverty, and humanitarian crisis. Identifying residual symptoms that persist after PM+ will assist future development of programs that could address lingering problems that people experience after receiving brief transdiagnostic programs.

## CONFLICT OF INTERESTS

The authors declare that there are no conflict of interests.

## ETHICS STATEMENT

The authors assert that all procedures contributing to this study comply with the ethical standards of the relevant national and institutional committees on human experimentation and with Helsinki Declaration of 1975, as revised in 2008.

### 1 PEER REVIEW

The peer review history for this article is available at https://publons.com/publon/10.1002/da.23221

## Data Availability

Requests for sharing of trial data used in this individual patient data meta‐analysis can be made to the corresponding author. Any sharing of data will be subject to obtaining appropriate agreements from the principle investigators or data custodians for each individual trial data set used in this study.
